# Curcumin Suppresses Intestinal Fibrosis by Inhibition of PPAR*γ*-Mediated Epithelial-Mesenchymal Transition

**DOI:** 10.1155/2017/7876064

**Published:** 2017-01-22

**Authors:** Su Xu, Bin Jiang, Hui Wang, Cunsi Shen, Hao Chen, Li Zeng

**Affiliations:** ^1^First Clinical Medical College, Nanjing University of Chinese Medicine, No. 138 Xianlin Avenue, Nanjing 210023, China; ^2^Department of Colorectal Surgery, The Yancheng Affiliated Hospital of Nanjing University of Chinese Medicine, No. 53 People's Road, Yancheng 224001, China; ^3^Department of Colorectal Surgery, The Third Affiliated Hospital of Nanjing University of Chinese Medicine, No. 1 Jinling Road, Nanjing 210001, China

## Abstract

Intestinal fibrotic stricture is a major complication of Crohn's disease (CD) and epithelial-to-mesenchymal transition (EMT) is considered as an important contributor to the formation of intestinal fibrosis by increasing extracellular matrix (ECM) proteins. Curcumin, a compound derived from rhizomes of* Curcuma*, has been demonstrated with a potent antifibrotic effect. However, its effect on intestinal fibrosis and the potential mechanism is not completely understood. Here we found that curcumin pretreatment significantly represses TGF-*β*1-induced Smad pathway and decreases its downstream *α*-smooth muscle actin (*α*-SMA) gene expression in intestinal epithelial cells (IEC-6); in contrast, curcumin increases expression of E-cadherin and peroxisome proliferator-activated receptor *γ* (PPAR*γ*) in IEC-6. Moreover, curcumin promotes nuclear translocation of PPAR*γ* and the inhibitory effect of curcumin on EMT could be reversed by PPAR*γ* antagonist GW9662. Consistently, in the rat model of intestinal fibrosis induced by 2,4,5-trinitrobenzene sulphonic acid (TNBS), oral curcumin attenuates intestinal fibrosis by increasing the expression of PPAR*γ* and E-cadherin and decreasing the expression of *α*-SMA, FN, and CTGF in colon tissue. Collectively, these results indicated that curcumin is able to prevent EMT progress in intestinal fibrosis by PPAR*γ*-mediated repression of TGF-*β*1/Smad pathway.

## 1. Introduction

Intestinal stricture is a common complication in Crohn's disease (CD) [1]. Over one-third of patients with CD require surgery to relieve obstruction due to fibrotic strictures [2]. As a consequence of chronic, transmural inflammation, intestinal fibrosis in CD is mainly characterized by overproduction and deposition of extracellular matrix (ECM) [3]. ECM-producing cells are mainly derived from mesenchymal cells such as fibroblasts, myofibroblasts, and smooth muscle cells. It has been proved that fibroblasts can transform from nonmesenchymal cells to mesenchymal cells [4], leading to occurrence of epithelial-to-mesenchymal transition (EMT) which is a key process in organ fibrosis.

EMT, a phenotypic transition of epithelial cell, is a complex and dynamic phenomenon that is accompanied by a loss of the epithelial cell hallmarks such as E-cadherin and an acquisition of the mesenchymal characterized proteins including *α*-smooth muscle actin (*α*-SMA), fibronectin (FN), and connective tissue growth factor (CTGF) [5]. It is now widely believed that EMT can induce the production of ECM and promote the formation of the intestinal fibrosis through direct and indirect mechanisms [6].

Several studies have demonstrated that EMT is triggered by transforming growth factor beta (TGF-*β*) [7, 8] and the TGF-*β*/Smad pathway plays a crucial role in promoting intestinal fibrosis [3, 9]. TGF-*β* has been seen as perhaps the best characterized promoter of EMT [10]. The resistance to tissue fibrosis in intestine, by either the loss of Smad3 or the increase of Smad7 expression, is shown by disrupting the TGF-*β*/Smads signaling pathway [11]. And the profibrogenic role of the TGF-*β*/Smad pathway in intestinal strictures in CD is supported by the expression of a decreased Smad7 and an increased p-Smad2/3 [12]. In TGF-*β* signaling, Smad2 and Smad3 associated with the TGF-*β* receptor complex are phosphorylated and then form a complex with Smad4 and translocate into the nucleus where it regulates specific transcription of target genes in conjunction with other nuclear cofactors [13]. However, several antifibrogenic molecules, for example, IL-10 and peroxisome proliferator-activated receptor *γ* (PPAR*γ*) [14], seem to interact with TGF*β*/Smad signaling pathway, which affects the fibrotic process.

PPARs are nuclear receptors considered as novel molecules involved in tissue fibrogenesis [15], regulating gene transcription by binding to retinoid X receptors (RXR) [16]. Three different isoforms of PPARs have been identified to be involved in several physiological processes including fibrosis [15, 17]. In particular, the PPAR*γ* isoform has been identified to distribute in the colorectal mucosa. In some past studies, PPAR*γ* activation seems to be strongly related to the TGF-*β*/Smads pathway and the stimulation of PPAR*γ* with specific ligands acts on the Smad3 pathway by antagonizing Smad3 or downregulating CTGF expression in human hepatic stellate cells and hypertrophic scar fibroblasts [16, 18]. Experimental studies have shown that the PPAR*γ* agonists attenuate fibrosis in various organs including lung, kidney, pancreas, and liver, and the PPAR*γ* antagonists, such as GW9662, abolish antifibrotic effects [19–23]. Despite these findings, both the underlying mechanisms for the regulatory effects of PPAR*γ* ligands on intestinal fibrosis and the specific role of PPAR*γ* in this process are unclear.

Curcumin [1,7-bis(4-hydroxy-3-methoxyphenyl)-1,6-heptadiene-3,5-dione] is an active component of turmeric derived from the rhizome of the plant* Curcuma longa* which is widely used as a coloring agent and spice in many foods all over the world, especially in India and China. Over the last 60 years, a variety of pharmacological properties of curcumin have been identified, including antioxidant, antibacterial, antifungal, antiviral, anti-inflammatory, antiproliferative, and anticarcinogenic properties [24–26]. Particularly, it is reported that curcumin has preventive and therapeutic significance in autoimmune diseases such as CD [24, 25]. Although some studies have suggested that curcumin has antifibrotic effect in liver, lung, and cystic fibrosis [27–30], the efficacy and mechanism of curcumin for intestinal fibrosis remain relatively limited.

In the previous study, curcumin has been reported to function as a PPAR*γ* agonist, enhancing the expression of PPAR*γ* [31], and to prevent the development of tissue fibrosis [27–30]. Here, we want to demonstrate that curcumin exerts inhibitory effect on intestinal fibrosis, which is dependent on activation of PPAR*γ* and subsequent occurrence of TGF-*β*/Smad signaling-mediated EMT.

## 2. Materials and Methods

### 2.1. Materials

Curcumin and 2,4,5-trinitrobenzene sulphonic acid (TNBS) were purchased from Sigma (USA); rosiglitazone was kindly provided by Huanghe Medical Share Co. Ltd. (China). Anti-E-cadherin was from Bioworld (USA) and ELISA kit of FN from Bluegene (USA); Anti-Smad2/3, anti-pSmad3, anti-PPAR*γ* were purchased from Cell Signaling Technology (USA); PPAR*γ* antagonist GW9662 was from Selleckchem (USA); IEC-6 cell line was obtained from American Type Culture Collection, ATCC. Dulbecco's modified Eagle's medium (DMEM) and foetal bovine serum (FBS) were purchased from Biological Industries (Israel) and all other chemicals were from AMRESCO (USA). Sprague-Dawley (SD) rats were purchased from Matter Technology (China).

### 2.2. Cell Culture and Induction of EMT In Vitro

IEC-6 cells were cultured in Dulbecco's modified Eagle's medium (DMEM) supplemented with 10% FBS in a 37°C incubator containing 5% CO_2_. For induction of EMT, the medium was replaced with DMEM with 0.5% FBS containing TGF-*β*1 (10 ng/mL) when the cells reached 50% confluency. The whole course lasts for 7 days and the medium was refreshed every 2 days [32].

### 2.3. Enzyme-Linked Immunosorbent Assay (ELISA)

ELISA was used to determine the level of FN in the supernatant of IEC-6 cells. The conditioned cultured supernatant was collected and centrifuged at 12,000 rpm for 5 min; then it was stored at −80°C for use. The commercial ELISA kits were used to detect FN. The protocols were performed according to the manufacturer instructions.

### 2.4. Western Blot Analysis

The IEC-6 cell lysates were sonicated and centrifuged at 14,000 rpm for 10 min at 4°C for preparing the loading protein samples. Protein concentration was determined by bicinchoninic acid assay. After the protein samples were subjected to sodium dodecyl sulfate polyacrylamide gel electrophoresis (SDS-PAGE), the separated protein was transferred to polyvinylidene difluoride (PVDF) membranes. Then the membranes were immersed in blocking buffer (5% nonfat dry milk containing 0.1% Tween 20) for 2 hours and washed 3 times with PBS-T completely, followed with incubation with the indicated primary antibodies overnight at 4°C. Finally, membranes were subsequently incubated with appropriate secondary antibody conjugated to horseradish peroxidase at room temperature for 30 min, and immunoreactive bands were detected by the enhanced chemiluminescence (ECL) detection system and photographed by a Bio-Rad image system (XRS+).

### 2.5. RT-qPCR

The mRNA expression was quantified in a 7500 fast real-time PCR system from ABI corporation. cDNAs and the specific primers were mixed with 2X iQ SYBR Green Supermix (Bio-Rad). The reaction parameters were 95°C for 3 min, 40 cycles of 95°C for 15 s and 60°C for 30 s, and 72°C for 5 min. PCR products were calculated by melting curves through fluorescence shift observed using a 7500 real-time PCR system (Applied Biosystems). The relative abundance of mRNAs was obtained using the comparative cycle threshold method and was normalized to the housekeeping control gene GAPDH, and ΔCT was calculated by subtracting the CT value of the GAPDH reference gene from that of each target gene. Results were expressed as fold changes (ΔΔCT) in the mRNA levels of a gene compared with the treated or untreated samples. The PCR primers are as follows: GAPDH, forward, 5-GAAGGTGAAGGTCGGAGTC-3, reverse, 5-GAAGATGGTGATGGGATTTC-3; PPAR*γ*, forward, 5-ACCACTCGCATTCCTTTGAC-3, reverse, 5-ATCGCACTTTGGTATTCTTGGAG-3; CTGF, forward, 5-ATGCTCGCCTCCGTCGCAG-3, reverse, 5-TCAAAGATGTCATTGTCCCCAG-3; E-cadherin, forward, 5-GCCAATCCTGATGAAATTGGAA-3, reverse, 5-CAGAACCACTGCCCTCGTAATC-3; *α*-SMA, forward, 5-ATAACATCAAGCCCAAATCTGC-3, reverse, 5-TTCCTTTTTTCTTTCCCAACA-3.

### 2.6. Immunofluorescence Stain

Rat IEC-6 cells were grown as monolayers on polycarbonate filters and on chamber slides. After the cells were treated by curcumin, the medium was discarded and the cells were fixed in 4% paraformaldehyde and blocked in CAS Block (Invitrogen) for 1 h at room temperature. IEC-6 cells were covered by the solution containing the anti-pSmad3 and anti-PPAR*γ* antibody, respectively, at 4°C overnight. Cells were next washed three times in PBS and stained with Cy-2-conjugated secondary antibody at room temperature for 2 hours. Stained cells were mounted with antifade mounting medium on slides and viewed using a fluorescence microscope.

### 2.7. Induction of Intestinal Colitis and Fibrosis in Rats

The TNBS-induced intestinal fibrosis was replicated in rats according the study [33]. Except the blank group, rats of other five groups were treated with TNBS to induce the intestinal fibrosis. In detail, the rats were anesthetized with pentobarbital sodium and received a weekly intracolonic instillation of 5% TNBS with a dose of 10 mg, 15 mg, 20 mg, 25 mg, and 30 mg on the 1st, 8th, 15th, 22th, and 29th day, respectively, plus 50% ethanol (0.8 mL) via a 2 mm diameter catheter inserted approximately 8 cm proximal to the anus. The blank control rats were given an equal volume of 50% ethanol by the same way. And the rats were held in a vertical position for 1 min after the intrarectal perfusion to ensure the distribution of TNBS within the entire colon and cecum [33]. Simultaneously, the TNBS-treated groups were given, by oral gavage, rosiglitazone (8 mg kg^−1^ day^−1^) or curcumin (50, 100, and 200 mg kg^−1^ day^−1^) from 1st day. And the disease activity index (DAI) was evaluated based on the change of rat weight, faeces, and fecal occult blood according the described methods to justify inflammatory condition in colon [34]. After 35-day curcumin treatment, all the rats were killed under anaesthesia. The colon of each mouse was rapidly excised and washed in phosphate-buffered saline (PBS), and the scores of colon macroscopic damage index (CMDI) of colonic lesions including adhesions, strictures, dilation, thickness, oedema/hyperaemia, and ulcers were evaluated according to the described methods [35]. Finally, the colon was cut into small sections. The tissue samples were then fixed in 10% buffered formaldehyde for histological analysis, but in 4% paraformaldehyde for immunohistochemistry analysis. The experiment was approved by the Animal Ethics Committees of Nanjing University of Chinese Medicine and performed strictly according to the NIH guide for the Care and Use of Laboratory Animals.

### 2.8. Histological Analysis of Intestinal Fibrosis

The large intestine specimens were washed and immediately immersed in 10% buffered formalin in PBS (pH 7.4) for fixation at room temperature and then embedded in paraffin. Sections were cut to a thickness of 4 *μ*m and stained with haematoxylin and eosin (H&E) or with Masson's trichrome for analyzing the fibrosis of tissue. The slides were then observed and photographed by a microscope. The score was valued based on the presence of ulcerations, the degree of inflammation, and the depth of the lesions and fibrosis according to the previous methods [35].

### 2.9. Immunohistochemistry

The large intestine tissues obtained as previously described were promptly fixed with 4% paraformaldehyde in PBS (pH 7.4), then dehydrated in gradient ethanol, and embedded in paraffin. The paraffin slides were deparaffinized to hydration and heated at 95°C in 0.01 M sodium citrate buffer (pH 6.0) for antigen repair. Then the slides were incubated in 3% hydrogen peroxide solution and 5% goat serum and rinsed in PBS. Thereafter, the solution containing polyclonal antibodies to TGF-*β*1, *α*-SMA, and E-cadherin was, respectively, dropped on the slides which were placed at 4°C overnight. The slides were then rinsed with PBS and incubated with second antibody labeled streptavidin-biotin-peroxidase. After being rinsed in PBS, the slides were incubated with 3, 3-diaminobenzidine-tetrahydrochloride (DAB). Finally, the slides were counterstained with Mayer's haematoxylin and observed by a fluorescent microscope.

### 2.10. Statistical Analysis

Experimental quantitative values were represented as the mean ± SD. One-way analysis of variance (ANOVA) with a post hoc Dunnett's *t*-test was used to compare the differences among the groups. A level of *p* < 0.05 was considered statistically significant.

## 3. Results

### 3.1. Curcumin Inhibited the Expression of *α*-SMA and Increased E-Cadherin in TGF-*β*1-Induced IEC-6 Cells

**Figure 1:**
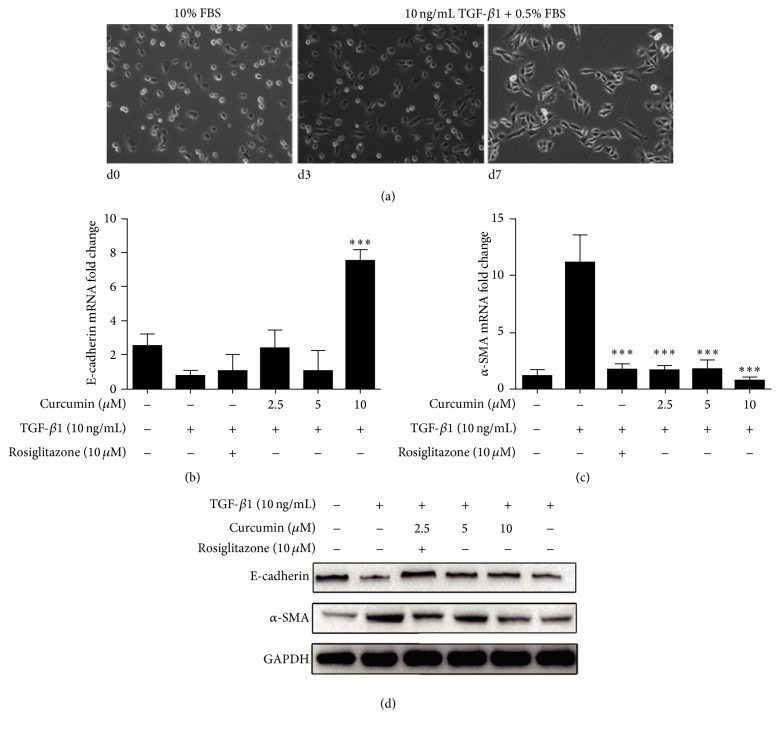
Curcumin inhibited the expression of *α*-SMA and increased E-cadherin in TGF-*β*1-induced IEC-6 cells. (a) IEC-6 cells were incubated with TGF-*β*1 for induction of EMT. (b, c) After stimulation by TGF-*β*1 (10 ng/mL), E-cadherin, and *α*-SMA of IEC-6 cells mRNA expressions were analyzed by RT-PCR. (d) After stimulation by TGF-*β*1 (10 ng/mL), protein expression of E-cadherin and *α*-SMA in IEC-6 cells were analyzed by Western blotting. Significance: ^*∗∗∗*^*p* < 0.001 compared with TGF-*β*1 group.

An EMT was induced in IEC-6 cells by TGF-*β*1. IEC-6 cells in DMEM medium containing 10% FBS displayed cuboidal morphology in the absence of TGF-*β*1. After exposure to medium DMEM plus 0.5% FBS with TGF-*β*1 (10 ng/mL) for 7 days, IEC-6 cells acquired a spindle-shaped morphology gradually (Figure 1(a)). E-cadherin and *α*-SMA expression were detected by both RT-PCR and Western blot. E-cadherin was prominent in normal IEC-6 cells. Curcumin only at 10 *μ*M upregulated E-cadherin mRNA expression (Figure 1(b)); however, at 2.5 *μ*M, 5 *μ*M, and 10 *μ*M, curcumin inhibited the *α*-SMA mRNA expression significantly compared with TGF-*β*1 induced group, so did the positive control of rosiglitazone treatment (Figure 1(c)). E-cadherin protein expression was inhibited obviously upon incubation with TGF-*β*1 and increased by curcumin at all three concentrations, while the *α*-SMA protein expression significantly was decreased under curcumin treatment compared with TGF-*β*1 induced group (Figure 1(d)).

### 3.2. Curcumin Inhibits Smad3 and Its Nuclear Translocation during EMT in TGF-*β*1-Induced IEC-6 Cells

**Figure 2:**
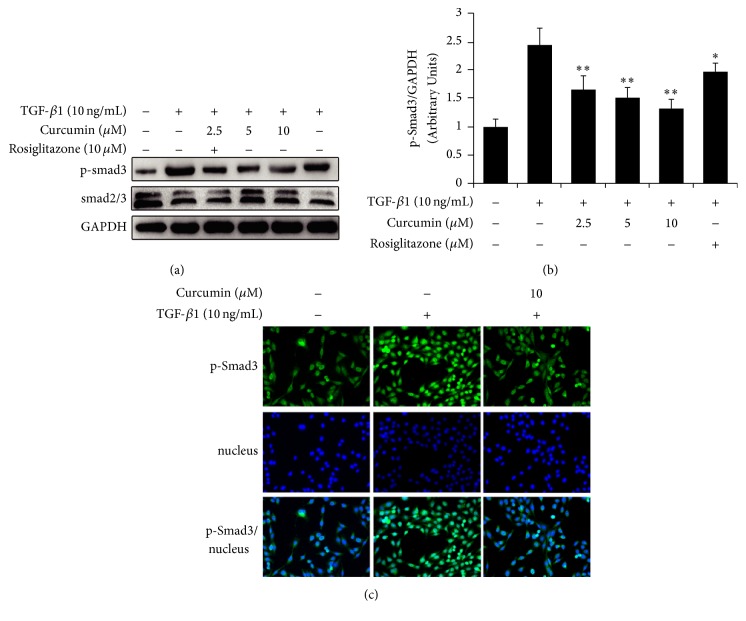
Effects of curcumin on Smad3 in IEC-6 cells with EMT induced by TGF-*β*1. IEC-6 cells were incubated with curcumin at the indicated doses for 24 hours followed by treatment with TGF-*β*1 for 7 days. (a) Western blotting analysis for Smad2/3 and p-Smad3 expression. (b) The ratio of grey values of p-Smad3/GAPDH bands, ^*∗*^*p* < 0.05, ^*∗∗*^*p* < 0.01, difference versus TGF-*β*1 group. (c) Immunofluorescence staining for nuclear translocation of pSmad3. Green indicates p-Smad3.

Upon TGF-*β*1 stimulation, Smad3 are phosphorylated and the expression of p-Smad3 is significantly more than the control without TGF-*β*1 stimulation in IEC-6 cells by Western blot analysis (Figure 2(a)). However, curcumin exerts distinct inhibition on high expression of p-Smad3 in a concentration-dependent manner (Figure 2(a)), and the quantitation of grey values of bands also shows the significant difference (*p* < 0.01) between the TGF-*β*1 induction and curcumin treatment (Figure 2(b)). Meanwhile, the immunofluorescence staining result also showed that TGF-*β*1 increased the expression of pSmad3; on the contrary, curcumin decreased the expression of pSmad3 and inhibited nuclear translocation of pSmad3 (Figure 2(c)).

### 3.3. Curcumin Promotes Activation of PPAR*γ* and Nuclear Translocation during EMT of TGF-*β*1-Induced IEC-6 Cells

**Figure 3:**
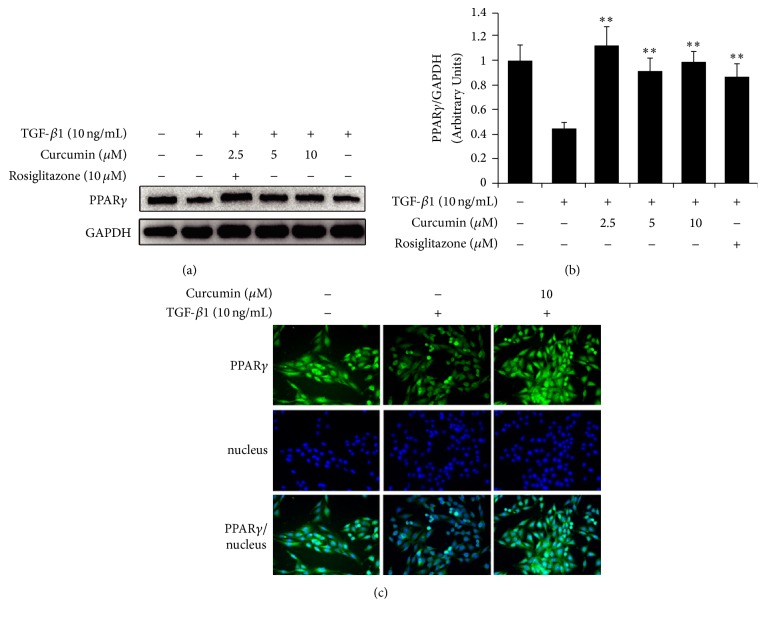
Curcumin reverses the reduction of TGF-*β*1-induced PPAR*γ* and promotes nuclear translocation of PPAR*γ*. IEC-6 cells were incubated with curcumin at the indicated doses for 24 hours or rosiglitazone for 1 hour followed by treatment with TGF-*β*1 for 7 days. (a) Western blotting analysis for PPAR*γ* expression in IEC-6 cells. (b) The ratio of grey values of PPAR*γ*/GAPDH bands, ^*∗∗*^*p* < 0.01, difference versus TGF-*β*1 group. (c) Immunofluorescence staining for nuclear translocation of PPAR*γ*. Green indicates PPAR*γ*.

Since curcumin inhibited TGF-*β*1-activated Smad3 in IEC-6 cells, it is necessary to understand whether the PPAR*γ*, an important TGF-*β*1/Smad3 pathway regulator, was involved. As expected, PPAR*γ* expression was downregulated by the induction of TGF-*β*1 in IEC-6 cells. However, pretreatment with curcumin for 24 hours reversed TGF-*β*1-induced PPAR*γ* inhibition, and 2.5 *μ*M curcumin shows the optimal reversal effect, as well as the agonist rosiglitazone (Figure 3(a)), and the quantitation of bands also shows the significant difference (*p* < 0.01) between the TGF-*β*1 induction and curcumin treatment (Figure 3(b)). At the meantime, we also detected expression of PPAR*γ* both in cytoplasm and in nuclei using immunofluorescence staining assay for analyzing PPAR*γ* translocation. And the results showed that curcumin increased the expression of PPAR*γ* and promoted the translocation of PPAR*γ* from the cytoplasm to the nucleus in TGF-*β*1-induced IEC-6 cells (Figure 3(c)).

### 3.4. PPAR*γ* Antagonist GW9662 Reverses TGF-*β*1-Induced EMT in IEC-6 Cells

**Figure 4:**
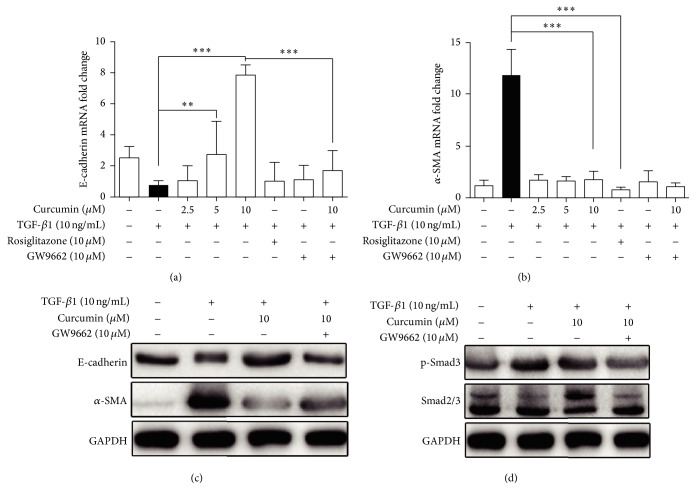
PPAR*γ* antagonist GW9662 reverses TGF-*β*1-induced EMT in IEC-6 cells. (a, b) mRNA expression of E-cadherin and *α*-SMA in IEC-6 cells was analyzed by RT-PCR at the presence of GW9662. ^*∗∗∗*^*p* < 0.001, ^*∗∗*^*p* < 0.01. (c, d) Protein expression of E-cadherin, *α*-SMA, smad2/3, and p-smad3 in IEC-6 cells was analyzed by Western blotting in the presence of GW9662.

To further demonstrate curcumin's effect on PPAR*γ* and PPAR*γ*-mediated pathway, the PPAR*γ* antagonist GW9662 was used to judge the effect of curcumin. As indicated in Figure 4(a), curcumin 5 *μ*M and 10 *μ*M significantly increased E-cadherin mRNA expression induced by 10 ng/mL of TGF-*β*1, which was completely blocked by 10 *μ*M antagonist GW9662. However, the significant decreased *α*-SMA mRNA expression induced by curcumin was not increased by 10 *μ*M GW9662 (Figure 4(b)). Seen from protein expression, curcumin promotion of E-cadherin in TGF-*β*1-induced IEC-6 cells was partially attenuated by GW9662 (Figure 4(c)), while curcumin inhibition of *α*-SMA protein was also partially reversed by GW9662 (Figure 4(c)). Furthermore, the trend by which curcumin inhibited pSmad3 protein expression in IEC-6 cells was reversed by GW9662 (Figure 4(d)). Taken together, all of the data indicated that curcumin inhibition of TGF-*β*1-induced EMT is dependent on PPAR*γ*.

### 3.5. Curcumin Inhibits TNBS-Induced Colitis Associated Intestinal Fibrosis in Rats

**Figure 5:**
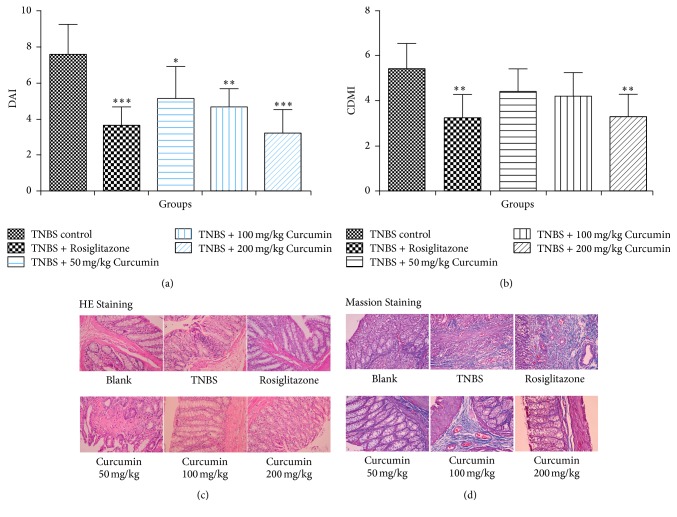
Curcumin inhibits TNBS-induced colitis associated intestinal fibrosis in rats. (a) The DAI scores of the colon macroscopic damage of the TNBS control group and the curcumin treatment groups with indicated doses. ^*∗*^*p* < 0.05, ^*∗∗*^*p* < 0.01, ^*∗∗∗*^*p* < 0.001 compared with TNBS control group. (b) The CDMI scores of the TNBS control group and curcumin treatment groups with indicated doses. ^*∗∗*^*p* < 0.01 compared with the TNBS control group. (c) A representative microscopic histology (HE staining) of the colon from five groups of rats. (d) Masson staining of colon tissues from five groups of rats.

The above in vitro data justified that curcumin could activate PPAR*γ* and subsequently inhibit TGF-*β*1-induced EMT in IEC-6 cells; the further question needs to be answered whether the results could be repeated in vivo. Thus, the TNBS-induced colitis associated intestinal fibrosis in rats was performed, and the related parameters such as E-cadherin, *α*-SMA, and PPAR*γ* were evaluated, as well as the DAI and CDMI scores in colon. Firstly, the DAI scores of curcumin treatment groups and TNBS model group were significantly increased over the score of blank control group (score: 0). Significant improvement was observed macroscopically in all three curcumin treatment groups, as the score of the three dose groups of curcumin is significantly less than the TNBS control group, and the improvement effect is dose-dependent (Figure 5(a)). Secondly, CDMI scores for evaluating colon lesions including adhesions, strictures, ulcers, and wall thickness revealed obvious difference between the TNBS-induced group and curcumin treatment groups. However, the statistically significant improvement was just observed in the high-dose curcumin treatment group followed with the score of this group (3.25 ± 0.97) significantly less than the TNBS control group (5.40 ± 1.02) (Figure 5(b)). Further pathological results of HE staining indicated that the dense cellular fibrosis was observed in the colonic submucosa in TNBS model rats, whereas just the mild fibrosis occurred in the submucosa in rats of curcumin treatment groups (100 and 200 mg/kg) with decreased inflammatory cells infiltration (Figure 5(c)). Additionally, histological findings of the colon tissues with Masson's trichrome staining showed that the severe collagen deposition in colon, observed in TNBS-induced rats, was undetected in rats with 200 mg/kg curcumin (Figure 5(d)).

### 3.6. Curcumin Inhibits Related Protein Involved in EMT in TNBS-Induced Colitis Associated Intestinal Fibrosis in Rats

**Figure 6:**
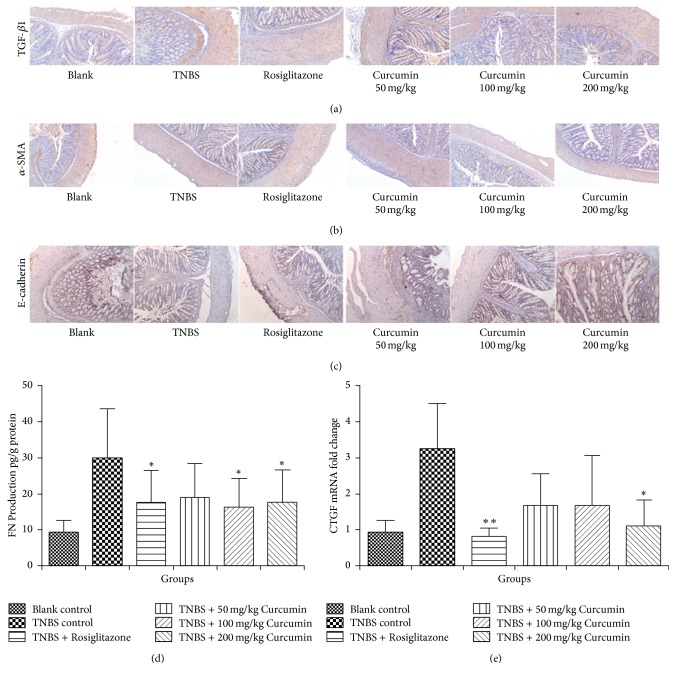
Curcumin inhibits related protein involved in EMT in TNBS-induced colitis associated intestinal fibrosis in rats. (a, b, c) Immunohistochemical analysis of TGF-*β*1, *α*-SMA, and E-cadherin in the colon tissue, the representative photos were selected. (d) Level of FN expression was detected by ELISA. ^*∗*^*p* < 0.05, compared with TNBS control group. (e) The expression of CTGF mRNA was analyzed by RT-PCR. ^*∗*^*p* < 0.05, ^*∗∗*^*p* < 0.01, compared with TNBS control group.

Besides the macroscopic and microscopic data, the related parameters involved in EMT, including TGF-*β*1, *α*-SMA, and E-cadherin, were determined by immunohistochemistry, as well as FN level and CTGF mRNA by ELISA and RT-PCR, respectively. A marked reduction of TGF-*β*1 staining was observed in the colon of all curcumin treatment groups compared to the colon of TNBS model group (Figure 6(a)), but no appreciable change was found in the expression of *α*-SMA (Figure 6(b)). Compared with the TNBS model group, both 100 and 200 mg/kg curcumin significantly increase the level of the immunostaining pattern of E-cadherin (Figure 6(c)). There was a significant decrease in the FN level (ELISA) after the treatment of curcumin (100 and 200 mg/kg/day). The expression of CTGF mRNA with a similar trend as the expression of FN was inhibited in the curcumin (200 mg kg^−1^ day^−1^) treatment group, compared with TNBS model group (Figure 6(e)).

### 3.7. Effect of Curcumin on PPAR*γ* Expression of TNBS-Induced Colitis Associated with Intestinal Fibrosis

**Figure 7:**
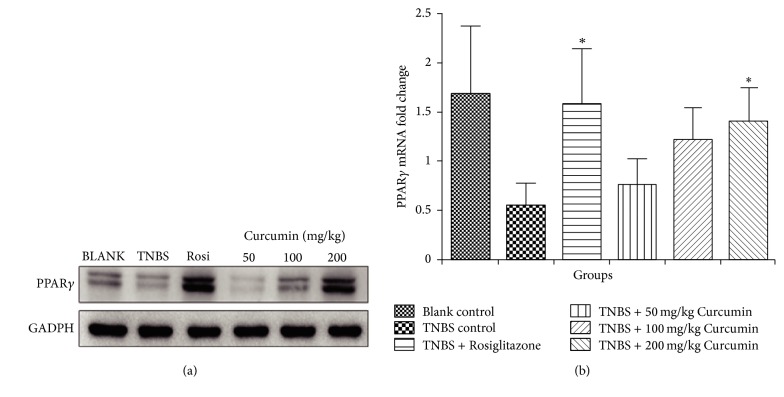
Effect of curcumin on PPAR*γ* expression of TNBS-induced colitis associated with intestinal fibrosis. (a) The expression of PPAR*γ* protein in the colon tissue of rats was determined by Western blotting. (b) The expression of PPAR*γ* mRNA in the colon tissue of rats was determined by RT-PCR. ^*∗*^*p* < 0.05, compared with TNBS control group.

To demonstrate the relationship between the PPAR*γ* and curcumin inhibition of EMT, the protein and mRNA level of PPAR*γ* were evaluated in the colon tissue by Western blot analysis and RT-PCR. As shown in Figure 7(a), the basal PPAR*γ* protein of colon tissue of blank group expresses more than that of the TNBS group with the weaker PPAR*γ* band. However, PPAR*γ* expression at 200 mg/kg group is more than both the blank and TNBS group, suggesting that curcumin could activate PPAR*γ*. And it was also demonstrated by the mRNA data of PPAR*γ*. Seen from Figure 7(b), curcumin promotes PPAR*γ* mRNA expression of colon tissue from intestinal fibrosis rat and a significant difference was calculated at 200 mg/kg group compared with TNBS control group.

## 4. Discussion

Nowadays, although the inflammatory PPAR*γ* pathogenesis of CD has been extensively studied and the therapeutic progress in the treatment of CD in the last twenty years, the main and specific cellular and molecular pathways leading to fibrosis remain to be identified. So far, there has not been an efficient and well-tolerated antifibrotic drug [36], and the incidence of intestinal fibrotic strictures in CD has not significantly changed. This implies that control of intestinal inflammation may not effectively affect the associated fibrotic process [14]. Curcumin, derived from the rhizome of the plant* Curcuma longa* often used as a kind of natural spice and common edible raw materials of pigment, has been used for treating IBD inflammation in clinic studies [37]. However, the inhibitory effect of curcumin on intestinal fibrosis has not been justified. A key finding of our study is the demonstration of the beneficial effects of curcumin on intestinal fibrosis and the role played by PPAR*γ* during EMT progress mediated by TGF-*β*/Smad signaling pathway.

Can curcumin inhibit EMT through the key TGF-*β*/Smad signaling pathway in intestinal fibrosis by activating PPAR*γ*? In order to answer this question, we used TGF-*β*1-induced EMT of IEC-6 cells model in vitro to verify the first step (Figure 1(a)). Suppression of E-cadherin is considered the earliest changes in TGF-*β*1-induced EMT [38] and *α*-SMA is an important marker of fibroblast contributing to the morphology of the epithelial cells changing. The expression of increased E-cadherin and decreased *α*-SMA indicated the reversed EMT in varying degrees after curcumin treatment (Figures 1(b) and 1(c)). Furthermore, curcumin increases the expression of PPAR*γ* and promotes nuclear translocation of PPAR*γ* in IEC-6 cells (Figure 3) and these results confirmed the previous reports that curcumin activated PPAR*γ* which was regarded as PPAR*γ* agonist [31, 39, 40].

In the second step, based on the results found above, the mechanisms underlying the effect of curcumin on EMT of IEC-6 cells were further investigated. TGF-*β*/Smads signaling is considered the most important pathway in EMT and most of the TGF-*β*1-induced EMT appears to be dependent on this signal pathway. The phosphorylation of Smad2 and Smad3 is an essential step in the signaling cascade. Studies suggested that TGF-*β*1 fibrotic events, as induction of CTGF and downregulation of E-cadherin, were critically dependent on Smad3, and Smad2 is involved in delayed events such as induction of MMP-2 and *α*-SMA requiring both Smad2 and Smad3 [41]. In our studies, we test the Smad2/3 and phosphorylated Smad3 treated by curcumin during the course of TGF-*β*1 induced EMT in IEC-6 cells and the results showed that phosphorylation of Smad3 was inhibited by the treatment of curcumin (Figure 2).

In order to investigate the further role of activated PPAR*γ* playing in TGF-*β*1/Smads signaling pathway, the third step, we used the PPAR*γ* antagonist GW9662 to further demonstrate it reversely. The results indicated that PPAR*γ* antagonist GW9662 reversed inhibition of nuclear translocation and the expression of E-cadherin and *α*-SMA although the phosphorylation of Smad3 seems not to be in TGF-*β*1-induced EMT in IEC-6 cells. Meanwhile, the mRNA of E-cadherin was reversed by GW9662, but not the mRNA of *α*-SMA. All of the mentioned above clued that PPAR*γ* mediates curcumin inhibition of EMT. However, it has been reported that curcumin could inhibit TGF-*β*1-induced EMT through PPAR*γ* pathway, but not Smad pathway in renal tubular epithelial cells, and possibly exert therapeutic effect in renal tubulointerstitial fibrosis [42]. Our results are consistent with this study on one hand, suggesting that curcumin inhibits intestinal fibrosis possibly by the same mechanism for renal fibrosis; on the other hand, Smad mediating curcumin inhibition of intestinal fibrosis also indicates that curcumin has distinctive effect on the different epithelial cells from different organ, finally leading to the different mechanisms.

At last, the conclusions we have obtained from in vitro studies were verified in vivo. In intestinal fibrosis model of rats, by oral curcumin, the levels of fibrogenic cytokines such as TGF-*β*1, *α*-SMA, E-cadherin, FN, and CTGF were decreased (Figure 6); antifibrogenic cytokine PPAR*γ* increased (Figure 7). The extent of intestinal fibrosis was improved (Figure 5).

In the previous researches, both antifibrogenic cytokine PPAR*γ* and TGF-*β*1/Smads signaling pathway were strongly related to the organ and tissue fibrosis [19–23, 43], while the relationship between PPAR*γ* and TGF-*β*1/Smads pathway in intestinal fibrosis seemed unclear. We had known that the stimulation of PPAR*γ* could interfere with the Smad3 pathway promoting TGF-*β*1 induced synthesis of collagen [15, 18], but we did not know that the process mentioned above was in a direct or an indirect way. In order to investigate this problem, we use the tool of PPAR*γ* antagonist GW9662. In our study, when GW9662 was added, the levels of E-cadherin and *α*-SMA changed; the most importantly, the levels of Smad2/3 and phosphorylated Smad3 of TGF-*β*1/Smads signaling pathway also changed. Based on the above appearance, we concluded that curcumin activated PPAR*γ* to influence the TGF-*β*1/Smads pathway.

In summary, our study showed that curcumin regulated the TGF-*β*1/Smads pathway by activating PPAR*γ* to improve the intestinal fibrosis. In conclusion, some of the molecular and histological findings of the present study reveal the prevention properties of curcumin against intestinal fibrosis by acting as PPAR*γ* agonist. Current studies indicate the role of curcumin in activating PPAR*γ* to intervene TGF-*β*1/Smads pathway on intestinal fibrosis. Further studies are needed to identify the specific molecular events. Since curcumin can be widely found in human food, it could be postulated that curcumin has a great potential as a novel antifibrogenic agent to be used in the treatment of fibrotic strictures in CD.

## 5. Conclusion

Curcumin inhibits TGF-*β*1-induced Smad-mediated EMT by activating PPAR*γ* in vitro and exhibits significant improved effect on the intestinal fibrosis induced by TNBS in rats.

## References

[1] Freeman H. J. (2003). Natural history and clinical behavior of Crohn's disease extending beyond two decades. *Journal of Clinical Gastroenterology*.

[2] Froehlich F., Juillerat P., Mottet C. (2008). Fibrostenotic Crohn's disease. *Digestion*.

[3] Speca S., Giusti I., Rieder F., Latella G. (2012). Cellular and molecular mechanisms of intestinal fibrosis. *World Journal of Gastroenterology*.

[4] Rieder F., Fiocchi C. (2008). Intestinal fibrosis in inflammatory bowel disease: progress in basic and clinical science. *Current Opinion in Gastroenterology*.

[5] Lee J. M., Dedhar S., Kalluri R., Thompson E. W. (2006). The epithelial-mesenchymal transition: new insights in signaling, development, and disease. *The Journal of Cell Biology*.

[6] Li Y., Dai F., Peng Q., Gan H. (2013). Progress in study on mechanism of intestinal fibrosis in inflammatory bowel disease. *Chinese Journal of Gastroenterology*.

[7] Xu J., Lamouille S., Derynck R. (2009). TGF-*β*-induced epithelial to mesenchymal transition. *Cell Research*.

[8] Miettinen P. J., Ebner R., Lopez A. R., Derynck R. (1994). TGF-beta induced transdifferentiation of mammary epithelial cells to mesenchymal cells: involvement of type I receptors. *The Journal of Cell Biology*.

[9] Wynn T. A. (2008). Cellular and molecular mechanisms of fibrosis. *Journal of Pathology*.

[10] O'Connor J. W., Gomez E. W. (2014). Biomechanics of TGF*β*-induced epithelial-mesenchymal transition: implications for fibrosis and cancer. *Clinical and Translational Medicine*.

[11] Latella G., Vetuschi A., Sferra R. (2009). Smad3 loss confers resistance to the development of trinitrobenzene sulfonic acid–*induced* colorectal fibrosis. *European Journal of Clinical Investigation*.

[12] Di Sabatino A., Jackson C. L., Pickard K. M. (2009). Transforming growth factor *β* signalling and matrix metalloproteinases in the mucosa overlying Crohn's disease strictures. *Gut*.

[13] Shi Y. G., Massagué J. (2003). Mechanisms of TGF-*β* signaling from cell membrane to the nucleus. *Cell*.

[14] Latella G., Sferra R., Speca S., Vetuschi A., Gaudio E. (2013). Can we prevent, reduce or reverse intestinal fibrosis in IBD?. *European Review for Medical and Pharmacological Sciences*.

[15] Rousseaux C., Desreumaux P. (2006). The peroxisome-proliferator-activated gamma receptor and chronic inflammatory bowel disease (PPAR*γ* and IBD). *Journal de la Société de Biologie*.

[16] Zhao C., Chen W., Yang L., Chen L., Stimpson S. A., Diehl A. M. (2006). PPAR*γ* agonists prevent TGF*β*1/Smad3-signaling in human hepatic stellate cells. *Biochemical and Biophysical Research Communications*.

[17] Houseknecht K. L., Cole B. M., Steele P. J. (2002). Peroxisome proliferator-activated receptor gamma (PPAR*γ*) and its ligands: a review. *Domestic Animal Endocrinology*.

[18] Zhang G., Cheng T., Zheng M. (2009). Activation of peroxisome proliferator-activated receptor-*γ* inhibits transforming growth factor-*β*1 induction of connective tissue growth factor and extracellular matrix in hypertrophic scar fibroblasts in vitro. *Archives of Dermatological Research*.

[19] Yang L., Stimpson S. A., Chen L., Harrington W. W., Rockey D. C. (2010). Effectiveness of the PPAR*γ* agonist, GW570, in liver fibrosis. *Inflammation Research*.

[20] Kawai T., Masaki T., Doi S. (2009). PPAR-*γ* agonist attenuates renal interstitial fibrosis and inflammation through reduction of TGF-*β*. *Laboratory Investigation*.

[21] Aoki Y., Maeno T., Aoyagi K. (2009). Pioglitazone, a peroxisome proliferator-activated receptor gamma ligand, suppresses bleomycin-induced acute lung injury and fibrosis. *Respiration*.

[22] Talukdar R., Tandon R. K. (2008). Pancreatic stellate cells: new target in the treatment of chronic pancreatitis. *Journal of Gastroenterology and Hepatology*.

[23] Chen H., He Y.-W., Liu W.-Q., Zhang J.-H. (2008). Rosiglitazone prevents murine hepatic fibrosis induced by *Schistosoma japonicum*. *World Journal of Gastroenterology*.

[24] Aggarwal B. B., Kumar A., Bharti A. C. (2003). Anticancer potential of curcumin: preclinical and clinical studies. *Anticancer Research*.

[25] Aggarwal B. B., Harikumar K. B. (2009). Potential therapeutic effects of curcumin, the anti-inflammatory agent, against neurodegenerative, cardiovascular, pulmonary, metabolic, autoimmune and neoplastic diseases. *International Journal of Biochemistry and Cell Biology*.

[26] Sharma R. A., Gescher A. J., Steward W. P. (2005). Curcumin: the story so far. *European Journal of Cancer*.

[27] Yao Q.-Y., Xu B.-L., Wang J.-Y., Liu H.-C., Zhang S.-C., Tu C.-T. (2012). Inhibition by curcumin of multiple sites of the transforming growth factor-beta1 signalling pathway ameliorates the progression of liver fibrosis induced by carbon tetrachloride in rats. *BMC Complementary and Alternative Medicine*.

[28] Punithavathi D., Venkatesan N., Babu M. (2000). Curcumin inhibition of bleomycin-induced pulmonary fibrosis in rats. *British Journal of Pharmacology*.

[29] Lipecka J., Norez C., Bensalem N. (2006). Rescue of ΔF508-CFTR (cystic fibrosis transmembrane conductance regulator) by curcumin: involvement of the keratin 18 network. *The Journal of Pharmacology and Experimental Therapeutics*.

[30] Nan Y.-M., Han F., Kong L.-B. (2011). Adenovirus-mediated peroxisome proliferator activated receptor gamma overexpression prevents nutritional fibrotic steatohepatitis in mice. *Scandinavian Journal of Gastroenterology*.

[31] Wang H.-M., Zhao Y.-X., Zhang S. (2010). PPAR*γ* agonist curcumin reduces the amyloid-*β*-stimulated inflammatory responses in primary astrocytes. *Journal of Alzheimer's Disease*.

[32] Flier S. N., Tanjore H., Kokkotou E. G., Sugimoto H., Zeisberg M., Kalluri R. (2010). Identification of epithelial to mesenchymal transition as a novel source of fibroblasts in intestinal fibrosis. *Journal of Biological Chemistry*.

[33] Zhu M. Y., Lu Y. M., Ou Y. X., Zhang H. Z., Chen W. X. (2012). Dynamic progress of 2,4,6-trinitrobenzene sulfonic acid induced chronic colitis and fibrosis in rat model. *Journal of Digestive Diseases*.

[34] Dieleman L. A., Pena A. S., Meuwissen S. G. M., Van Rees E. P. (1997). Role of animal models for the pathogenesis and treatment of inflammatory bowel disease. *Scandinavian Journal of Gastroenterology, Supplement*.

[35] Videla S., Vilaseca J., Medina C. (2006). Selective inhibition of phosphodiesterase-4 ameliorates chronic colitis and prevents intestinal fibrosis. *Journal of Pharmacology and Experimental Therapeutics*.

[36] Fiocchi C., Kay Lund P. (2011). Themes in fibrosis and gastrointestinal inflammation. *American Journal of Physiology—Gastrointestinal and Liver Physiology*.

[37] Taylor R. A., Leonard M. C. (2011). Curcumin for inflammatory bowel disease: a review of human studies. *Alternative Medicine Review*.

[38] Willis B. C., Borok Z. (2007). TGF-*β*-induced EMT: mechanisms and implications for fibrotic lung disease. *American Journal of Physiology—Lung Cellular and Molecular Physiology*.

[39] Chen A., Xu J. (2005). Activation of PPAR*γ* by curcumin inhibits Moser cell growth and mediates suppression of gene expression of cyclin D1 and EGFR. *American Journal of Physiology - Gastrointestinal and Liver Physiology*.

[40] Li R., Wang Y., Liu Y. (2013). Curcumin inhibits transforming growth factor-*β*1-induced EMT via PPAR*γ* pathway, not smad pathway in renal tubular epithelial cells. *PLoS ONE*.

[41] Phanish M. K., Wahab N. A., Colville-Nash P., Hendry B. M., Dockrell M. E. C. (2006). The differential role of Smad2 and Smad3 in the regulation of pro-fibrotic TGF*β*1 responses in human proximal-tubule epithelial cells. *Biochemical Journal*.

[42] Li R., Wang Y., Liu Y. (2013). Curcumin inhibits transforming growth factor-*β*1-induced EMT via PPAR*γ* pathway, not Smad pathway in renal tubular epithelial cells. *PLoS ONE*.

[43] Kapoor M., McCann M., Liu S. (2009). Loss of peroxisome proliferator-activated receptor *γ* in mouse fibroblasts results in increased susceptibility to bleomycin-induced skin fibrosis. *Arthritis and Rheumatism*.

